# Anomaly Detection for Internet of Things Time Series Data Using Generative Adversarial Networks With Attention Mechanism in Smart Agriculture

**DOI:** 10.3389/fpls.2022.890563

**Published:** 2022-06-06

**Authors:** Weijun Cheng, Tengfei Ma, Xiaoting Wang, Gang Wang

**Affiliations:** ^1^School of Information Engineering, Minzu University of China, Beijing, China; ^2^Key Laboratory of Mining Disaster Prevention and Control, Shandong University of Science and Technology, Qingdao, China

**Keywords:** anomaly detection, smart agriculture, time series data, deep learning, generative adversarial network, attention mechanism

## Abstract

More recently, smart agriculture has received widespread attention, which is a deep combination of modern agriculture and the Internet of Things (IoT) technology. To achieve the aim of scientific cultivation and precise control, the agricultural environments are monitored in real time by using various types of sensors. As a result, smart agricultural IoT generated a large amount of multidimensional time series data. However, due to the limitation of applied scenarios, smart agricultural IoT often suffers from data loss and misrepresentation. Moreover, some intelligent decision-makings for agricultural management also require the detailed analysis of data. To address the above problems, this article proposes a new anomaly detection model based on generative adversarial networks (GAN), which can process the multidimensional time series data generated by smart agricultural IoT. GAN is a deep learning model to learn the distribution patterns of normal data and capture the temporal dependence of time series and the potential correlations between features through learning. For the problem of generator inversion, an encoder–decoder structure incorporating the attention mechanism is designed to improve the performance of the model in learning normal data. In addition, we also present a new reconstruction error calculation method that measures the error in terms of both point-wise difference and curve similarity to improve the detection effect. Finally, based on three smart agriculture-related datasets, experimental results show that our proposed model can accurately achieve anomaly detection. The experimental precision, recall, and F1 score exceeded the counterpart models by reaching 0.9351, 0.9625, and 0.9482, respectively.

## Introduction

Nowadays, Internet of Things (IoT) technology has been obtained rapidly developments, as a paradigm, to drive the evolution of modern industries and smart cities. As for serious challenges in environmental pollution, energy depletion, and water shortage in the whole world, there is an urgent need for the agriculture industry to move toward digitalization (Cao et al., 2021). To address these challenges, smart agriculture solutions based on real-time monitoring and decision-making have been received increasing attention. Smart agriculture is a deep combination of IoT technology and modern agriculture, which mainly takes modern agriculture as an application scenario and applies IoT technology to achieve a goal of scientific cultivation and precise control (Farooq et al., 2019).

**Figure 1:**
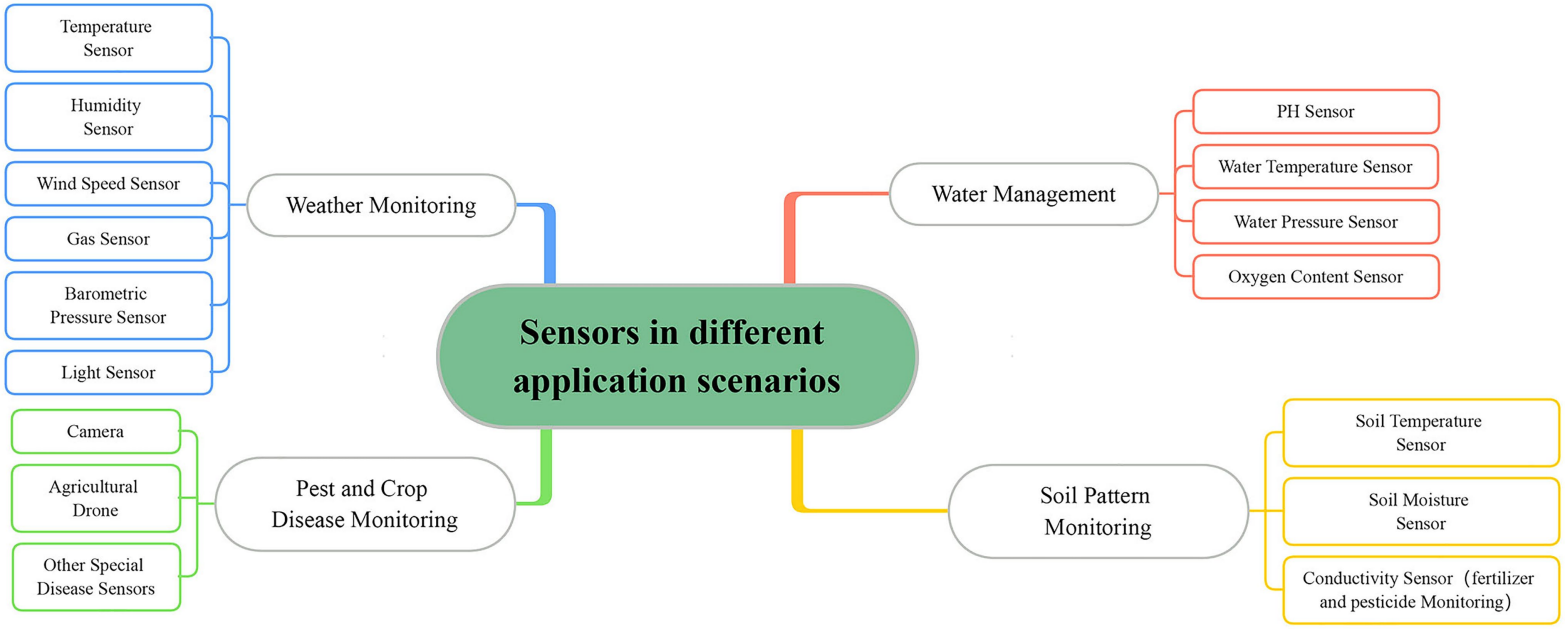
Sensors in different application scenarios.

For smart agriculture IoT systems, automated management and smart decision of IoT applications are driven by the detailed analysis of data (Cao et al., 2021). These data are collected by a large number of various types of sensors and provide information about different environmental conditions. Thus, environmental monitoring and data analysis play an important role in increasing crop yields. The sensors in different application scenarios are shown in Figure 1. However, IoT devices in smart agriculture are usually exposed to harsh environments and are highly susceptible to damage due to cost control (Rafii and Kechadi, 2019; Abdallah et al., 2021). In addition, the heterogeneous nature of network devices makes it difficult to design protocols, and the transmission of data is easily compromised (Pundir and Sandhu, 2021). Poor communication quality can lead to data loss and misrepresentation. Increasingly complex IoT systems bring technical complexity and therefore make the design of privacy and security mechanisms more difficult. This can also expose the network to attacks that could lead to data tampering (Abdallah et al., 2021). Missing or misrepresented data is significantly different from normal data in the time series data collected by the sensors (Moso et al., 2021). These can be considered as anomalies in the data (Adkisson et al., 2021). Moreover, IoT applications also require an algorithm to analyze these data to facilitate intelligent decision-making. By analyzing the data in detail, the intelligent system can make the most efficient resource scheduling to increase crop yield. Testing for crop growth patterns can help reduce soil depletion, and different weather and soil conditions can affect irrigation decisions (Vilenski et al., 2019; Vyas and Bandyopadhyay, 2020; Garg et al., 2021). The main idea of data analysis in the smart agriculture scenario is to analyze various sensor data, and the analysis results can reflect the changes in the environment (Khalil et al., 2021). In particular, data that differ from normal data due to environmental changes can be also designated as anomalies. Therefore, anomaly detection has become an important work of smart agricultural IoT.

The data collected by smart agriculture IoT sensors is mainly called stream data, also called time series data. They are a series of infinite data points with a timestamp T . The purpose of time series anomaly detection is to find anomaly points or anomaly subsequences in a time series. In previous years, machine learning-based data mining techniques have been evaluated and achieved high performance in anomaly detection (Nassif et al., 2021; Pang et al., 2021). Due to the specificity of time series, their data sets lack labeling information. Therefore, it is usually treated as unsupervised machine learning. Unsupervised tasks do not require expert knowledge and can automatically adapt to data changes (Yu et al., 2021). Different environmental indicators generate different time series data. Multiple time series form multidimensional time series data, which is the main data format in smart agriculture. There are potential correlations between the different dimensions of these data, which should be considered primarily. However, existing unsupervised machine learning methods cannot handle the non-linearity of potential correlations in multidimensional time series (Li et al., 2019). Up to date, with the increasing number and types of sensors, smart agriculture generated a large amount of time series data, and there exist two challenges in anomaly detection. One is that the amount of monitored variables and data points is exploding, and the other is that there are potential correlations and time dependencies between multidimensional variables. They lead to high-dimensional and heterogeneous time series data features, which cannot be accomplished by machine learning-based anomaly detection models (Yang et al., 2021). Thus, it is necessary to find some new research approaches to solve these emerging problems of anomaly detection in smart agriculture.

In recent years, deep learning has been proposed for anomaly detection, and most of them are reconstruction-based models. The general process of reconstruction-based anomaly detection is that a model is employed to learn the distribution of normal data, and then the trained model is used to reconstruct the data to be measured. The error between the reconstructed data and the original data is used to determine if the data is anomalous. Reconstruction-based anomaly detection models can model large-scale data and capture potential correlations between multidimensional data. Among them, generative adversarial networks (GAN) work well (Goodfellow et al., 2014). GAN generally contain generator and discriminator. The generator can generate samples, and the discriminator can determine whether the input sample is the original sample or the sample generated by the generator. The generator wants to generate samples that are closest to the original samples to fool the discriminator. The discriminator wants to accurately determine whether the sample is a real sample or not. The learning ability of the model is continuously improved by the adversarial learning of both. GAN was initially introduced to anomaly detection to solve problems related to image data (Schlegl et al., 2017; Zenati et al., 2018a). With the growth of the number and dimensionality of time series data, GAN was introduced to time series data anomaly detection due to its superiority in processing high-dimensional data. GAN-based anomaly detection belongs to the reconstruction-based anomaly detection models, in which GAN is used to learn the distribution of normal data. A trained GAN to reconstruct anomaly data will produce large reconstruction errors. Finally, the anomaly score is used to determine whether the test sample is anomalous, where the anomaly score mainly includes the reconstruction error.

Li et al. (2019) and Bashar and Nayak (2020) introduced the general GAN into the anomaly detection model for time series data. The goal of these algorithms is to detect time series data anomalies quickly and accurately by GAN. Generally, there are two main types of anomalies: one is data loss or data misrepresentation caused by equipment failure or network anomalies, and the other is data anomalies that do not conform to the potential correlation of normal data distribution. However, since the generator input of GAN is random normal data, this brings inconvenience to the calculation of reconstruction error. The calculation of each reconstruction error requires finding the optimal normal data corresponding to the reconstructed samples, which needs the inversion of the generator. This leads to a large computational cost and may also degrade the detection results. Some works in the field of anomaly detection have focused more on changes in model structure, but there have been few improvements to the way errors are calculated. Most studies considered only a single computational method, and the point-wise difference calculation was widely adopted (Li et al., 2019; Bashar and Nayak, 2020; Geiger et al., 2020). This does not exactly fit the time series data format and sometimes does not conform to the true definition of error. Time series data is a series of data points that can form a smooth curve. For the curve as a form of data, the curve similarity should be considered as an error measure.

Motivated by the above observation, we focus on the anomaly detection of multidimensional time series data, which is generated from different sensor data in smart agricultural systems. In this paper, we propose a new GAN-based anomaly detection method. In particular, for generator inversion, an encoder–decoder architecture is designed. In this architecture, we introduce an attention mechanism that can effectively improve the reconstruction effect. Then, a new reconstruction error calculation is provided. The point-wise difference and curve similarity are jointly considered as reconstruction errors, which makes the error definition more realistic and improves detection performance. Finally, we conduct experiments using three data sets related to smart agriculture and specialize the model parameters according to the data set characteristics. The experimental results show that our approach outperforms the other four counterpart anomaly detection methods.

## Related Work

With the rapid development of computer technology, researchers began to experiment with computer technology to solve anomaly detection. Hawkins (1980) had a widely accepted explanation of anomalies, namely, “in a given data set, anomalous data are that part of the data that is significantly different from the majority of the data.” Current anomaly detection methods can be broadly classified into proximity-based methods, probability-based methods, prediction-based methods, and deep learning-based methods (Aggarwal, 2017). These methods except deep learning are called traditional methods. They used statistical measures to calculate the correlation between the data records. These techniques assumed that the time series is linear and follows a known statistical distribution, which makes them inapplicable to many practical problems (Adhikari and Agrawal, 2013). As the volume and dimensionality of data grow, more deep learning algorithms have been proposed for anomaly detection on complex data. Deep learning-based anomaly detection methods have advantages over these methods in characterizing multidimensional time series data and are more helpful in solving practical problems.

Generally, time series data anomaly detection algorithms are divided into two steps. The data are modeled by different data structures and then the degree of deviation of the test data from the normal data is evaluated based on different forms of metrics (e.g., distance-based and density-based). Data with excessive deviations are judged to be abnormal. The deep learning-based anomaly detection is similar to the above process. Neural networks are used in the data representation phase to learn the data distribution, and reconstruction-based methods are applied in the anomaly calculation phase to measure error. Since the data structure learns the normal data distribution, there will be a large reconstruction error using this model to reconstruct abnormal data. In recent years, there has been an increasing number of studies using GAN for anomaly detection. As a result, a state-of-the-art survey of the anomaly detection for GAN is discussed in the following section.

### GAN for Image Anomaly Detection

AnoGAN (Schlegl et al., 2017) was the first work that applied GAN for image anomaly detection. The model was trained with normal data and the final anomaly scores were obtained by calculating errors in the trained generator and discriminator. The reconstruction error was calculated in the generator to calculate the error more efficiently. However, this computation requires finding the inverse mapping from the data space to the latent space and is not synchronized with the training. It can lead to extremely high error computation time. Zenati et al. (2018a) proposed an efficient GAN-based anomaly detection to solve the above problem. They added an extra encoder to GAN to avoid looking for latent vector at each detection. The calculation of the anomaly error was the same as the AnoGAN. Skip-GANomaly proposed by Akçay et al. (2019) introduced an architecture of skip connection to improve image reconstruction. The model improved image reconstruction but did not perform well on all data sets, which was limited by unstable training. Zenati et al. (2018b) pointed out that AnoGAN was inappropriate for real-time anomaly detection or larger data sets. They proposed a bi-directional GAN for image anomaly detection, which simultaneously trained the inverse mapping through an encoder network. The model contained three discriminators which effectively improved training stability.

### GAN for Time Series Anomaly Detection

The achievements of GAN in image anomaly detection have attracted the attention of researchers, and have been introduced into time series anomaly detection. Li et al. (2018) proposed a GAN-based anomaly detection method (GAN-AD) for time series data, which was used to detect possible anomalous behaviors in complex networks. To capture the correlation of time series data, Long Short-Term Memory networks (LSTM) were used as the basic model to learn normal data distribution patterns. For the evaluation of the error, since the output of the discriminator indicated whether the sample is false or not, it was used directly as the anomaly score to find the anomaly. Li et al. (2019) later extended their study to use a vanilla GAN model to capture multivariate time series model distributions and detect anomalies using reconstruction errors and discriminator outputs. Bashar and Nayak (2020) improved on AnoGAN (Schlegl et al., 2017) and proposed an anomaly detection algorithm for time series data. The model used a convolutional neural network (CNN) as the basic network to capture the correlation between variables. Both of the above models can learn the time correlation of time series data and effectively detect anomalies. However, they also need to find the inverse mapping from the real space to the latent space, which requires an inversion of the generator resulting in a longer computation time.

To address this problem, Geiger et al. (2020) proposed TadGAN based on Zenati et al. (2018a). This model introduced cycle-consistent GAN architectures, which allowed the generator to compute the reconstruction error directly without finding the inverse mapping and reducing the computation time. For the calculation of the anomaly score, the combination of point-wise difference and discriminator was typically considered as the anomaly score. However, using point-wise difference measures alone does not exactly fit the time series data characteristics. The time series can form different smoothing curves, and the shape differences between these curves should be equally considered in the reconstruction error calculation. The TadGAN used curve similarity as a form of calculation of reconstruction error. However, they studied the point-wise difference and curve similarity separately and did not consider them together to meet a realistic definition. Apart from this, the existing articles are insufficient for the study of reconstruction errors.

### Anomaly Detection in Smart Agriculture

Research on deep learning-based anomaly detection for IoT systems has yielded excellent results, some of which have been introduced into smart agriculture to address emerging challenges. Most of the research on smart agriculture has focused on the field of anomaly detection in agricultural images, such as the identification of pests and crop diseases. TPest-RCNN proposed by Li et al. (2021) aimed to identify whitefly and thrips in greenhouses. The model was trained on a set of pest images captured by a flytrap and used a transfer learning strategy to achieve improved detection. Liu and Wang (2020) optimized the feature layer of Yolo V3 model by using the image pyramid to achieve multi-scale feature detection and improved the detection accuracy and the speed of Yolo V3 model. Experiments showed that the model can accurately and quickly detect the location and category of tomato pests and diseases.

For time series data generated in agricultural IoT systems, some researchers have focused on anomaly detection of sensor network data. Several papers have offered specifics on anomalies in smart ecosystems (Cook et al., 2019; Hasan et al., 2019; Park et al., 2021). In smart agriculture scenarios, agricultural IoT devices are often exposed to harsh conditions that can lead to failure of the device itself, compromised communications, or malicious attacks, which can lead to data anomalies. Adkisson et al. (2021) proposed an anomaly detection model for smart farming using an unsupervised autoencoder machine learning model. The model used an autoencoder to encode and decode the data, and anomalous data generated a high reconstruction loss value. Ultimately, the test data was determined to be anomalous based on a threshold value. Abdallah et al. (2021) applied autoregressive integrated moving average (ARIMA) and LSTM model to a smart agricultural system and specialized models based on sensor constraints. The transfer learning strategies were introduced into the models to improve the prediction. Anomaly detection for time series data can also be applied in crop harvesting. Moso et al. (2021) proposed a powerful ensemble-based approach for anomaly detection, which was mainly used for data streams generated in smart agriculture. This technology can be applied to crop data sets and identify anomalies that affect crop harvest.

In summary, time series anomaly detection based on deep learning has obtained excellent results, and GAN for anomaly detection has been continuously explored. Various deep learning models have been introduced to solve the problem of time series data for smart agriculture. As the amount and dimensionality of data increase, existing smart agriculture anomaly detection models are unable to handle the data. GAN has been introduced to various fields to process multidimensional time series data with better results. Therefore, we introduce GAN into smart agriculture for anomaly detection and specialize model structure for smart agriculture data characteristics. However, the existing studies of GAN for anomaly detection are limited by the problem of generator inversion and the reconstruction error is calculated in a simple way. To address the problem of GAN for anomaly detection, we design a new architecture and an error calculation method to improve the anomaly detection performance.

## Proposed Approach

In this section, we first describe a novel GAN-based anomaly detection model and focus on how it uses an adversarial learning architecture by considering the dependencies between time series data. Then, the internal detailed architecture of the model is shown, which includes the encode–decoder in the generator and the structure of the discriminator. These designs have strong relevance to the goals of improving reconstruction effects and reducing error computation time. To better learn the data distribution, a multi-channel attention mechanism is embedded in the encoder and decoder, which can further improve the reconstruction effect. Finally, we introduce a new error calculation method in this model, which can describe the errors more rationally and improve the detection results effectively.

The core idea of the reconstruction-based anomaly detection method is to encode a data point (time series data in this model) and then decode the encoded data point to reconstruct the data. Anomalous data loses a lot of information during the encoding–decoding process, because what the model should learn is how to reconstruct normal data. Thus, a normal trained model cannot reconstruct abnormal data in the same way as normal data. Large reconstruction errors will arise in the process of reconstructing anomalous data. This means that the reconstructed data has a large difference from the original data. In this paper, GAN model is used to model the data in an attempt to learn the normal distribution of the data.

The basic task of anomaly detection is to identify whether the data to be tested conforms to the distribution of normal data, and data that do not conform to the normal distribution are defined as anomalous (Chalapathy and Chawla, 2019; Kwon et al., 2019). In this work, the completed trained GAN is used for anomaly detection. The test samples are processed in the same data processing manner and then fed into the model in an attempt to reconstruct them. The anomaly score is calculated using a jointly trained generator and discriminator, which consists of the output of the discriminator and the reconstruction error of the generator. For reconstruction errors, we use a new calculation to detect potential anomalies in the data (more details will be described in “Anomaly Detection”).

### The Proposed GAN Framework

**Figure 2:**
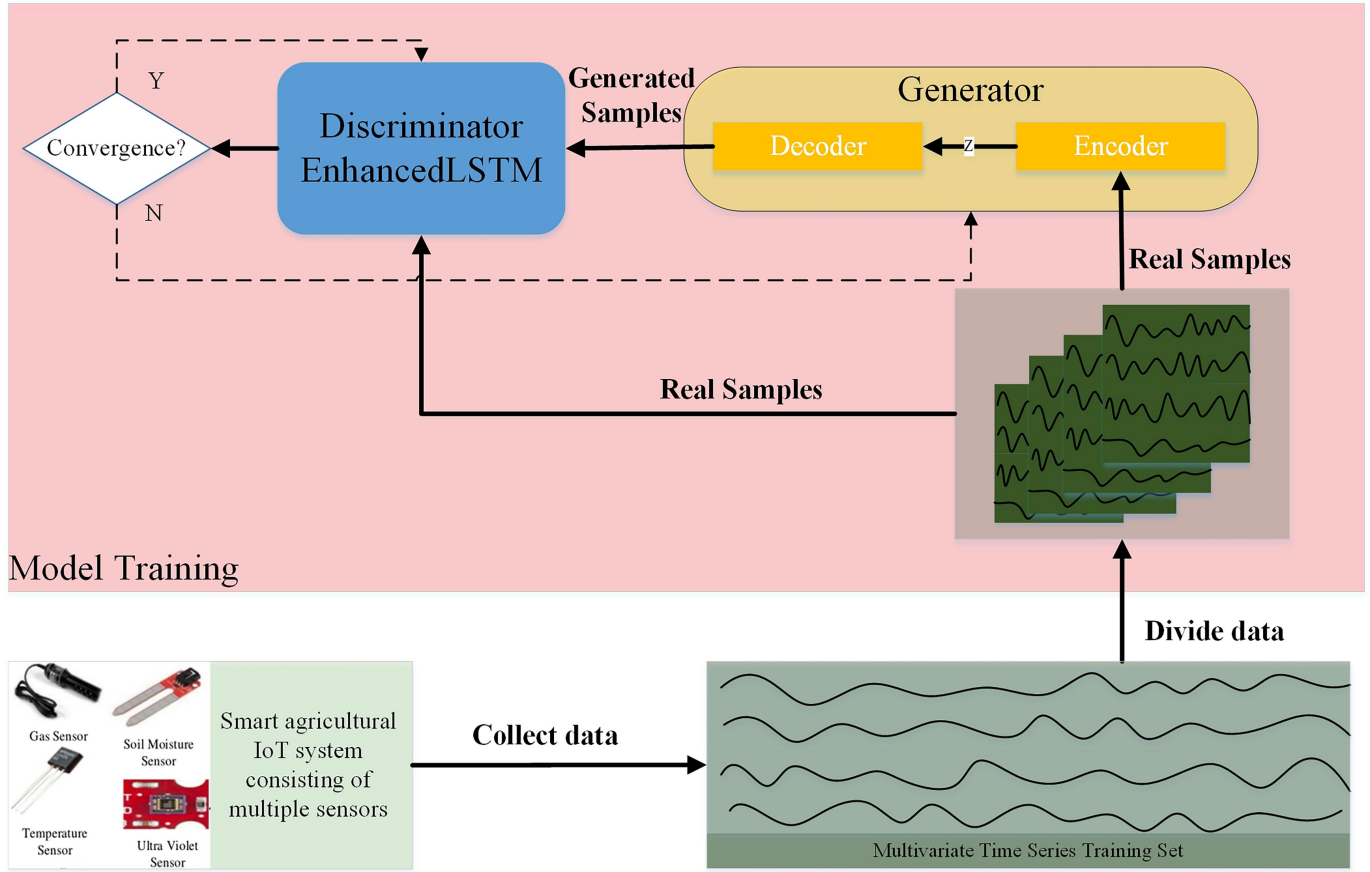
Overall framework of GAN.

The general architecture of our proposed model is shown in Figure 2. The first objective of this model is to learn the normal distribution of a given data set by means of adversarial training. Previous studies have taken random normal vectors in the latent space Z and inputted them into the generator for training (Li et al., 2019; Bashar and Nayak, 2020). The trained generator is able to implicitly capture the multivariate distribution of the training data and learn the mapping of random data to normal data. However, the error between the reconstructed time series data and the real data needs to be calculated in anomaly detection. In order to reconstruct the data, it is necessary to find the random normal data corresponding to the reconstructed data at each calculation. This process requires the inversion of the generator and is time-consuming and computationally resource intensive. To solve this problem, an encoder–decoder architecture is designed as a generator, in which the encoder learns the mapping of normal data to latent vector in the latent space and optimizes the computation time of reconstruction errors.Sensors in smart agricultural systems perform continuous measurement tasks to detect changes in the environment. Therefore, they generate a large amount of multivariate time series data. We use LSTM as the basic model of generator and discriminator to deal with complex multidimensional time series data. For the characteristics of multidimensional time series data, the data streams are not processed separately. The entire data set is processed concurrently to capture potential interactions between variables. Multivariate time series data are divided into subsequences that are fed into the model through a sliding window mechanism. We set the window size as the super parameter of the model to determine the optimal window length, which can capture the data distribution in different situations according to the characteristics of different data sets. Here, the window size is set as


$$s_{w}=30\times i,i=1,2,\cdots 10.$$

The data first needs to be preprocessed before training. The multivariate time series data $\Phi \subset R^{T\times N}$ of the length T and number N of variables are partitioned into a training set $\Phi_{train}\subseteq R^{T_{1}\times N}$ , a validation set $\Phi_{validation}\subseteq R^{T_{2}\times N}$ and a test set $\Phi_{test}\subseteq R^{T_{3}\times N}$ . Noted that the training set data must be all normal data. Next, the training data set $\Phi_{train}\subseteq R^{T_{1}\times N}$ is divided into a series of subsequences $X_{train}=\left\{X_{train}^{i}\mathrm{,i}=1,2,\cdots m\right\}\subseteq R^{S_{w}\times N}$ using a sliding window of size $s_{w}$ , where $X_{train}$ denotes $X_{t-s_{w}:t}$ . Given the step size $s_{t}$ , the number of subsequences can be calculated by $m=\left[\left(T_{1}-s_{w}\right)∕s_{t}\right]+1$ . Similarly, the validation set $\Phi_{validation}\subseteq R^{T_{2}\times N}$ can be partitioned into a series of subsequences $X_{validation}=\left\{X_{validation}^{j},j=1,2,\cdots n\right\}\subseteq R^{S_{w}\times N}$ , where $\;n=\left[\left(T_{2}-s_{w}\right)∕s_{t}\right]+1$ . Subsequences in the validation data set are marked to indicate whether the sequence is abnormal or not (1 means normal, 0 means abnormal). The test set is handled exactly in the same way as the validation set.

Model training is performed after the data preprocessing is completed. The distribution of the data is learned by the GAN model in adversarial training. In our model, the mapping functions of the X and Z domains are learned as ε:X→Z and G:Z→X , respectively. X is the input data, which represents the training samples ${\left\{\left(x_{i}^{1\ldots N}\right)\right\}}_{i=1}^{t}\in X$ given by the model. Z is the vector in latent space and the encoder learns the mapping ε:X→Z to encode the input data as a latent vector. The mapping G:Z→X is learned by the decoder, which reconstructs the vector in latent space to the input data. With the above two mapping functions, we can achieve the data reconstruction: $x_{i}\to \varepsilon \left(x_{i}\right)\to G\left(\varepsilon \left(x_{i}\right)\right)\approx x_{i}$ . These two mapping functions are obtained by adversarial learning methods, and together they form the generator of the GAN architecture.

The generator tries to deceive the discriminator by generating the real sample through the encoder–decoder architecture so that the discriminator judges the generated data as the real sample. To ensure that the distribution pattern of normal data is learned by the model, we make sure that the training data are all normal during the training phase. Unlike the general GAN that inputs the variables in latent space to the generator, the normal sample $x_{i}$ after data segmentation processing is directly fed into the generator and the two mapping functions ε and G mentioned above learn the mapping patterns of the two stages, respectively. The samples are reconstructed as much as possible to the original samples after two mappings. Both the generator output $\;G\left(\varepsilon \left(x_{i}\right)\right)$ and the original data $x_{i}$ are then sent to the discriminator to distinguish whether they are generated data or not. The generator tries to generate the same samples as the original data, and the discriminator tries to distinguish the real samples from the generated samples. This process is similar to the one in which the generator *G* uses the discriminator *D* as an adversary (Goodfellow et al., 2014). Adversarial training of both *G* and *D* continuously improves their performance until a set number of iterations is reached or the model converges. After continuous iterations of adversarial learning, the generator implicitly learns the normal data distribution and the discriminator can distinguish the real data from the generated data. The overall loss function in this process is mainly adversarial loss.

#### Adversarial Loss

Both the generator and the discriminator try to optimize the competing loss functions during training. Thus, the optimization process can be considered as a minimax game problem. During the game, the generator tries to minimize the loss to make the generated sample as close as possible to the original sample. The discriminator tries to maximize the loss to distinguish the real samples from the generated samples. The adversarial loss of the training process is defined as follows:


(1) $$L_{adv}=E_{X\sim pX}\left[\log\left(D\left(X\right)\right)\right]+E_{X\sim pX}\left[\log\left(1-D\left(G\left(\varepsilon \left(X\right)\right)\right)\right)\right]$$

where D(X) is the discriminator output, $\;E_{X\sim pX}$ represents the true sample sampled from the real space, log(D(X)) means that the original sample is expected to be judged as true by the discriminator, and $\log\left(1-D\left(G\left(\varepsilon \left(X\right)\right)\right)\right)$ means that the generated sample is expected to be considered false.

#### Feature Loss

GAN may lead to training instability when both the generator and the discriminator try to optimize the losses. To solve this problem, we introduced the feature matching proposed by Salimans et al. (2016) and used this loss function to stabilize the model training. It is defined by the following equation:


(2) $$L_{fea}=E_{X\sim pX}{\left\|f\left(X\right)-f\left(G\left(\varepsilon \left(X\right)\right)\right)\right\|}_{2}$$

where f(∗) is the output of the last layer of the discriminator, and the loss is $L_{2}$ norm of (X) and f(G(ε(X))) .

#### Mapping Loss

The goal of the model is to learn two mappings to reconstruct the sample. However, relying only on adversarial loss does not guarantee that a single original sample $x_{i}$ can be mapped to the latent vector $z_{i}$ and thus reconstructed as ${\hat{x}}_{i}$ . To reduce the search space in the mapping process, we minimize the $L_{2}$ norm of residuals of the original and reconstructed samples. Its loss can be calculated as


(3) $$L_{map}=E_{X\sim pX}{\left\|X-G\left(\varepsilon \left(X\right)\right)\right\|}_{2}$$

The generator tries to minimize the loss function, and the final overall loss function is obtained by combining (1), (2), and (3), as


(4) $$L_{G}=\lambda_{a}L_{adv}+\lambda_{f}L_{fea}+\lambda_{m}L_{map}$$

where $\lambda_{a}$ , $\lambda_{f,}\;\mathrm{and}\;\lambda_{m}$ represent the weights of each loss function, respectively.

The generator is trained directly using the adversarial loss in an attempt to maximize the following adversarial loss:


(5) $$L_{D}=E_{X\sim pX}\left[\log\left(D\left(X\right)\right)\right]+E_{X\sim pX}\left[\log\left(1-D\left(G\left(\varepsilon \left(X\right)\right)\right)\right)\right]$$

The set loss function will be optimally searched by employing stochastic gradient descent (SGD). After continuous iterative adversarial learning, the discriminator and generator performance are gradually improved. When the set epoch or loss function convergence is reached, the GAN model can learn the distribution of normal data. After that, anomaly scores can be designed based on the model output to detect anomalies (described in Section “Anomaly Detection”).

### The Architecture of Generator and Discriminator

**Figure 3:**
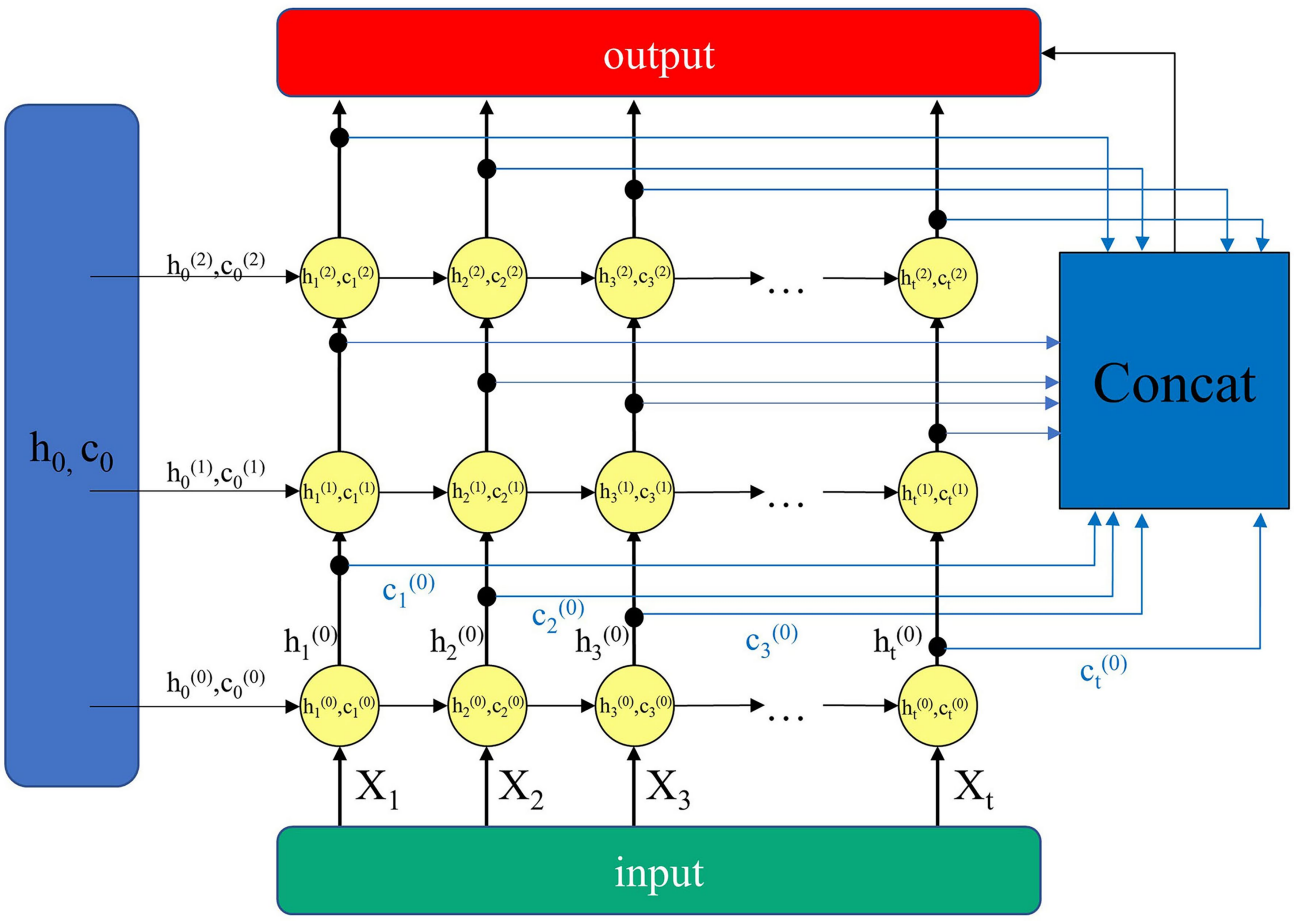
Enhanced LSTM structure.

To improve the reconstruction effect, we optimize the design of the generator for time series data, and the basic model of the generator and discriminator of GAN is designated as LSTM. Inspired by the Jiang (2020), the LSTM module is improved into the Enhanced LSTM structure, which consists of multiple LSTM structures, as shown in Figure 3. It shows in detail the internal structure of the 3-layer Enhanced LSTM we introduced. The horizontal direction is the time step of the LSTM, that is, the time window size. The vertical is the number of LSTM layers, so that $h_{t}^{\left(0\right)}$ and $c_{t}^{\left(0\right)}$ are the hidden cell state and memory state of the first LSTM layer at moment t, respectively. The general LSTM passes the hidden cell state $h_{i}$ and the memory state $c_{i}$ horizontally to the LSTM cell at the next moment and passes $h_{i}$ to the next LSTM layer in vertical direction.

Unlike the general stacked LSTM structure, the Enhanced LSTM binds both the hidden cell state and the memory state of each LSTM cell layer at a certain moment. It has the advantage of making full use of the hidden cell state and memory state of the current layer. The hidden cell state and the memory state of all layers except the current layer are used as the auxiliary input. This can improve the learning capability of the model network for time series data.

Recently, the attention mechanism has been widely applied in various research areas of neural networks. The attention mechanism allows the importance of different features to the final effect to be calculated, enabling the model to give higher weights to features that are beneficial to the outcome. In addition, the attention mechanism has a high degree of correctness and interpretability. RAIM (recurrent attentive and intensive model; Xu et al., 2018) was a model including an attention mechanism, which used multi-channel attention to improve the prediction of the model. Hashimoto et al. (2021) introduced RAIM into GAN to detect time series anomalies generated by semiconductor sensors. To this end, we consider a multi-channel attention mechanism, and an attention module is connected before both the encoder and the decoder.

**Figure 4:**
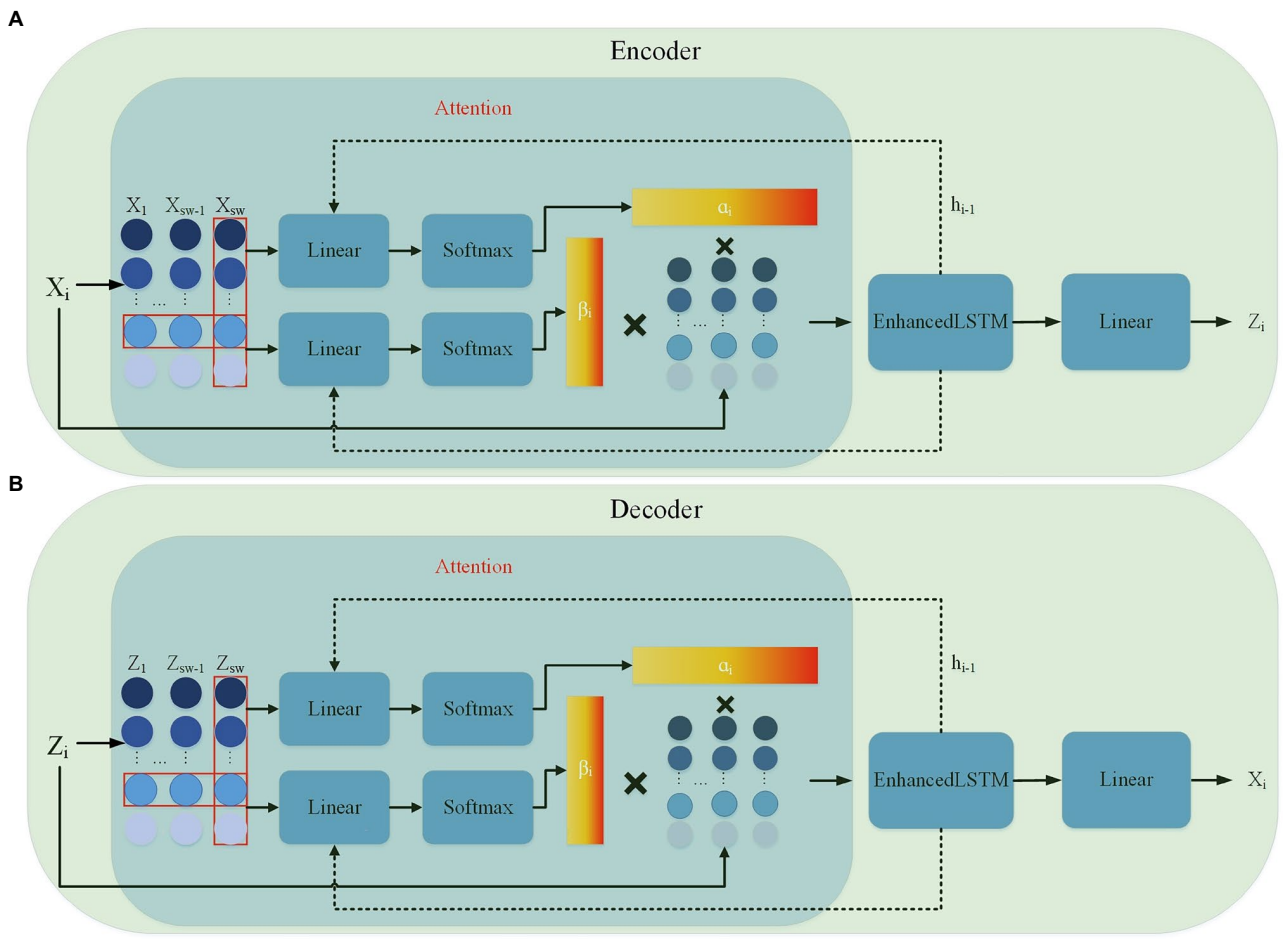
Encoder–decoder internal detailed structure **(A)** encoder structure; **(B)** decoder structure.

The multi-channel attention module is divided into two stages, which can adaptively give different weights to multidimensional variables. The encoder and decoder structures are shown in Figures 4A,B, respectively. The first stage performs the attention calculation in the time dimension. This mechanism based on the features extracted from the past sequences calculates the importance of different time steps of the input sequence. The input data $X=\left\{x_{t},t=1,\cdots ,T\right\}$ is a series of time series data of length T , where the data $x_{t}\in {\mathbb{R}}^{d}$ at time t is an d -dimensional vector. Let the i -th subsequence be $X_{i}\in {\mathbb{R}}^{s_{w}\times d}$ , when *X* is split by a sliding window $s_{w}$ (Hashimoto et al., 2021). Then the importance $a_{ij}$ of the time dimension is calculated by the following equation:


(6) $$S_{i}^{time}=\tanh\left(W_{h}^{a}\cdot h_{i-1}+X_{i}^{T}w_{x}^{a}+b^{a}\right)$$

(7) $$a_{ij}=\frac{\exp\left(S_{ij}^{time}\right)}{\Sigma_{j^{\prime }=1}^{S_{w}}\exp\left(s_{ij^{\prime }}^{time}\right)},j=1,2,\cdots ,S_{w}$$

where $W_{h}^{a}\epsilon {\mathbb{R}}^{s_{w}\times \left|h\right|}$ and $w_{x}^{a}\epsilon {\mathbb{R}}^{d\times 1}$ are the weighting matrices, $\;b^{a}\epsilon {\mathbb{R}}^{s_{w}\times 1}$ is the learning parameter through the attention mechanism, and $h_{i-1}$ is the hidden state vector extracted from the Enhanced LSTM in the previous time step.

The second stage performs attention calculation in the feature dimension. Within the same time step, different weights are given according to the importance of different dimensional features, and the importance $b_{ij}$ of each feature is calculated by the following equations:


(8) $$S_{i}^{feature}=\tanh\left(W_{h}^{\beta }\cdot h_{i-1}+X_{i}^{T}w_{x}^{\beta }+b^{\beta }\right)$$

(9) $$b_{ij}=\frac{\exp\left(S_{ik}^{feature}\right)}{\Sigma_{k^{\prime }=1}^{d}\exp\left(s_{ik^{\prime }}^{feature}\right)},k=1,2,\cdots ,d$$

where $W_{h}^{\beta }\epsilon {\mathbb{R}}^{d\times \left|h\right|}$ and $w_{x}^{\beta }\epsilon {\mathbb{R}}^{s_{w}\times 1}$ are the weighting matrices, $\;b^{\beta }\epsilon {\mathbb{R}}^{d\times 1}$ is the learning parameter through the attention mechanism, and $h_{i-1}$ is the hidden state vector extracted from the Enhanced LSTM at the previous time step. After a two-stage attention mechanism, the model can capture important features more accurately.

The attention mechanism calculates the importance of the features and time of the input multidimensional time series data and weights them with the input to obtain a new input. The Enhanced LSTM captures the correlation and time dependence between the input data weighted by the attention mechanism. In the encoder, the Enhanced LSTM input is the original sample after weighting and the output is the feature vector. After that, the linear layer independently maps each feature vector as a latent vector each time. The decoder then reconstructs the latent vector into the original data by the same process. The discriminator is a simple Enhanced LSTM architecture, which is mainly used to distinguish the input samples as the real samples or the reconstructed samples. The performance of the discriminator and generator is gradually improved by adversarial learning.

### Anomaly Detection

**Figure 5:**
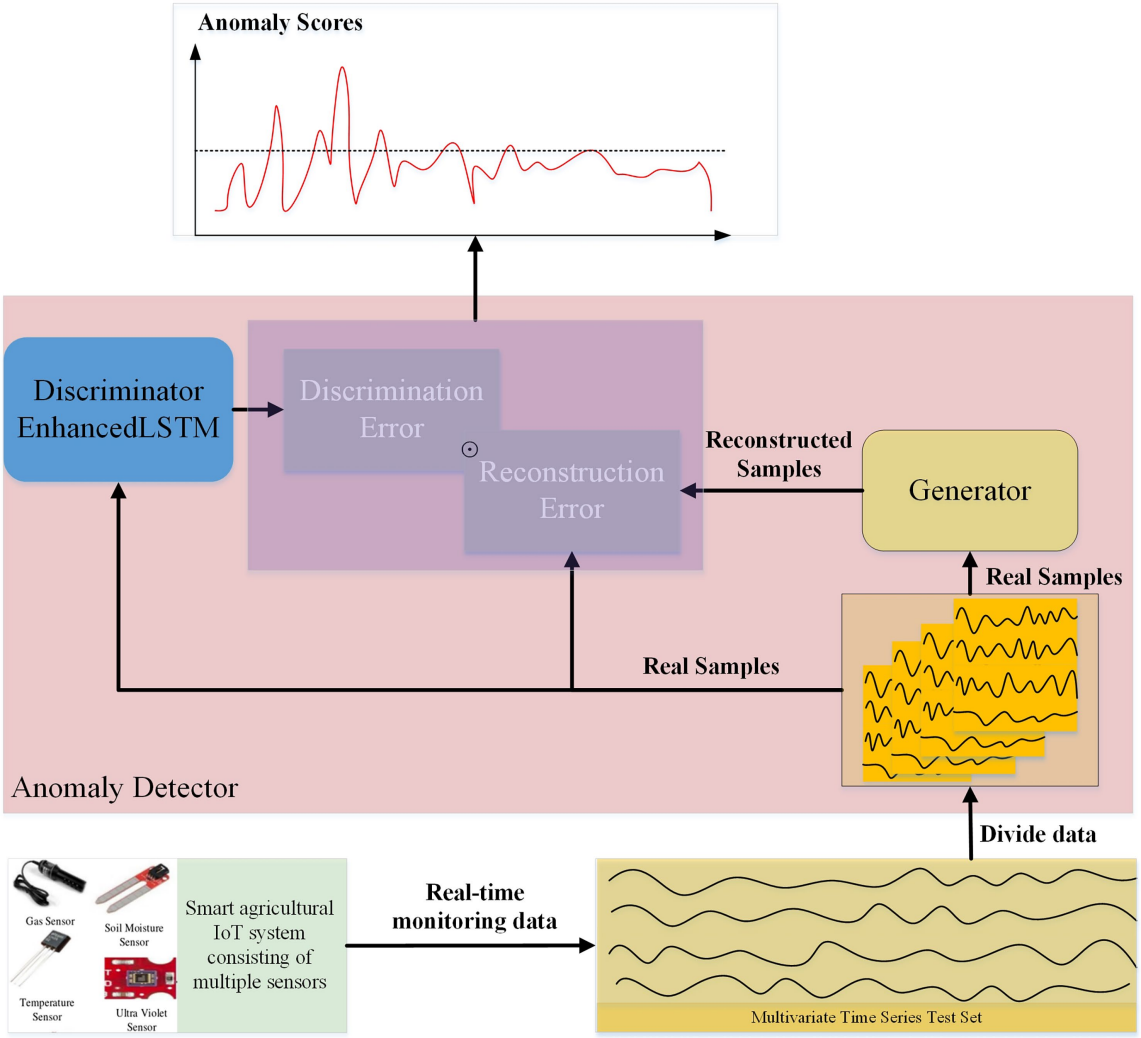
Anomaly detection.

Our proposed model has been iteratively trained to learn the distribution pattern of normal data. The GAN model has the advantage of training a generator and a discriminator together, both of which can output metrics to help identify anomalies. The anomaly detection process is shown in Figure 5. The labeled test set data are divided into subsequences according to time windows using the same method as the training set data. The segmented time series data $X_{test}=\left\{X_{test}^{j},j=1,2,\cdots n\right\}\subseteq R^{S_{w}\times N}$ will be binary classified. Each subsequence is determined to be normal (close to the normal data distribution) or abnormal (deviating from the normal data distribution) based on a threshold. We try different thresholds by using empirically determined threshold intervals and finally determine the threshold that results in optimal anomaly detection.

Reconstruction error is a measure of the difference between the true sample and the reconstructed sample. The ordinary generator only learns the mapping G:Z→X from random normal data in latent space to normal data, but there is no inverse mapping $G^{-1}:X\to Z$ . That is to say, it is necessary to find the optimal latent vector z∈Z , such that the sample G(z) reconstructed by the generator is closest to the test sample $x_{test}^{j}$ in terms of distribution pattern. This process is the inversion of the generator. It needs to be further trained for the test sample to find the optimal latent vector, which generates the reconstruction sample with minimum error. The general procedure is to randomly sample $z_{1}\in Z$ in the latent space and feed it into the generator to obtain the fake generative sequence $G\left(z_{1}\right)$ . After that, the loss function is defined for the generated samples and the best latent vector z is found by gradient update in successive iterations. The degree of similarity between the generated sample of the latent vector and the original sample determines the accuracy of the reconstruction error calculation.

In this paper, the generator has learned how to map the normal data in real space to the latent vector in latent space and then decode the latent vector back to the normal data. For the reconstructed samples, its corresponding latent vector is obtained by simply feeding it into the encoder without inversion. To improve the reconstruction effect and constrain the search domain, we add a new loss function (equation (3)). The reconstruction error can be obtained after the test data set is reconstructed by the generator. We combine two different error calculations to define the error more realistically.

The most intuitive way to measure the error is to use the point-wise difference, which directly calculates the difference between the corresponding points within each time step of the two series data. The error of the test data set at moment *t* is calculated as follows:


(10) $$l_{d}=\overset{n}{\underset{i=1}{\sum }}\left|x_{t}^{test,i}-G\left(\varepsilon \left(x_{t}^{test,i}\right)\right)\right|$$

where $x_{t}^{test,i}\epsilon R^{n}$ is the measured value of *i*-th variables at moment t .

**Figure 6:**
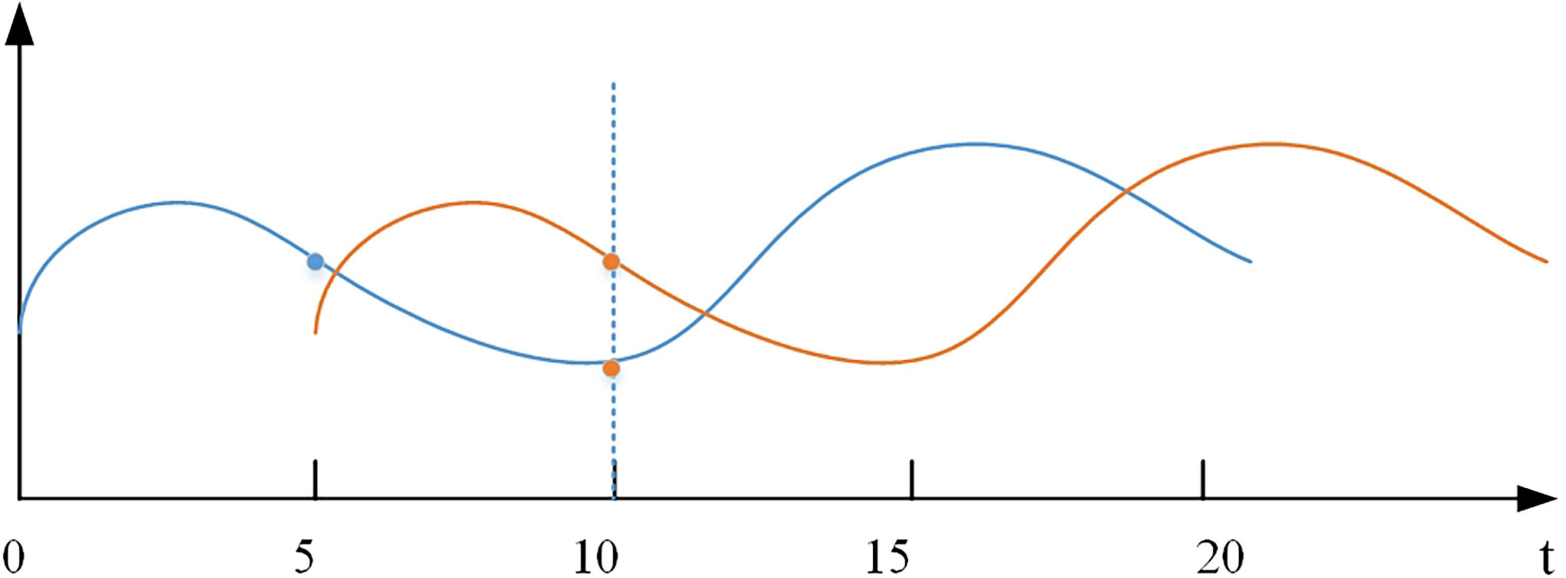
Time shift issue of time series.

Time series data is a series of data points that make up a smooth curve. For this feature, we introduce the dynamic time warping (DTW) algorithm (Berndt and Clifford, 1994), which calculates the optimal match between two time series data and measures whether the two curves are similar in shape. This algorithm can solve the time shift issue of time series. As shown in Figure 6, there are two curves with the same shape, but they are not synchronized in time steps. In the actual error calculation, this should be determined as a low error. However, using a point-wise difference at the 10^th^ time step leads to a larger error value. After the accumulation of multiple time steps, the error value may reach a level that affects the detection results. Based on this case, the DTW algorithm is introduced to measure the error more rationally. For the original subsequence $X=\left(x_{t},x_{t+1},\ldots ,x_{t+s_{w}-1}\right)$ and the reconstructed subsequence $\hat{X}=\left({\hat{x}}_{t},\;{\hat{x}}_{t+1},\ldots ,\;{\hat{x}}_{t+s_{w}-1}\right)$ , we define the matrix $W\epsilon R^{2*s_{w}\times 2*s_{w}}$ , let the (*i,j*)-th element $w_{k}$ represent the distance between $x_{i}$ and ${\hat{x}}_{j}$ . We want to find the warp path $w^{*}=\left(w_{1}\cdot w_{2},\cdots ,w_{k}\right)$ that defines the minimum distance between the two curves, subject to boundary conditions at the start and end, as well as constraints on continuity and monotonicity (Geiger et al., 2020). The two curve distances are defined as follows:


(11) $$S_{t}=W^{*}=DTW\left(X,\;\hat{X}\right)=\min\left[\frac{1}{k}\sqrt{\overset{k}{\underset{k=1}{\sum }}w_{k}}\right]$$

The final reconstruction error is given by using (10) and (11) as:


(12) $$L_{R}=\alpha L_{d}+\beta S_{t}\;$$

where α and β are the coefficients of the two reconstructed calculated values, which are the empirical values that make the experimental effect optimal.

During the training process, the main goal of the discriminator is to distinguish real samples from the generated samples and the output $L_{D}$ (between 0 and 1) can be regarded as a parameter to determine whether the sequence is a real sample (close to 1) or a fake sample (close to 0). Thus, the output of the discriminator can be used as a measure of the anomaly score. The reconstruction error and the discriminator output are considered together as the final anomaly score. However, the reconstruction error and discriminator output cannot be simply used because a larger reconstruction error with a smaller discriminator output can lead to a very high anomaly score. The above problem is solved by using numerical normalization. The normalized result is calculated by the following formula to obtain the final anomaly score.


(13) $$A\left(x\right)=\tau L_{R}+\left(1-\tau \right)L_{D}$$

where τ determines the relative importance of the two indicators (default value is 0.5).

Metrics, such as the precision of anomaly detection, can be calculated from the labeled test set data. Thresholds taken from the empirically determined threshold interval are used for anomaly detection. Different thresholds are obtained for different data sets, which results in optimal detection accuracy. Our proposed method is summarized in Algorithm 1.

**Algorithm 1:**
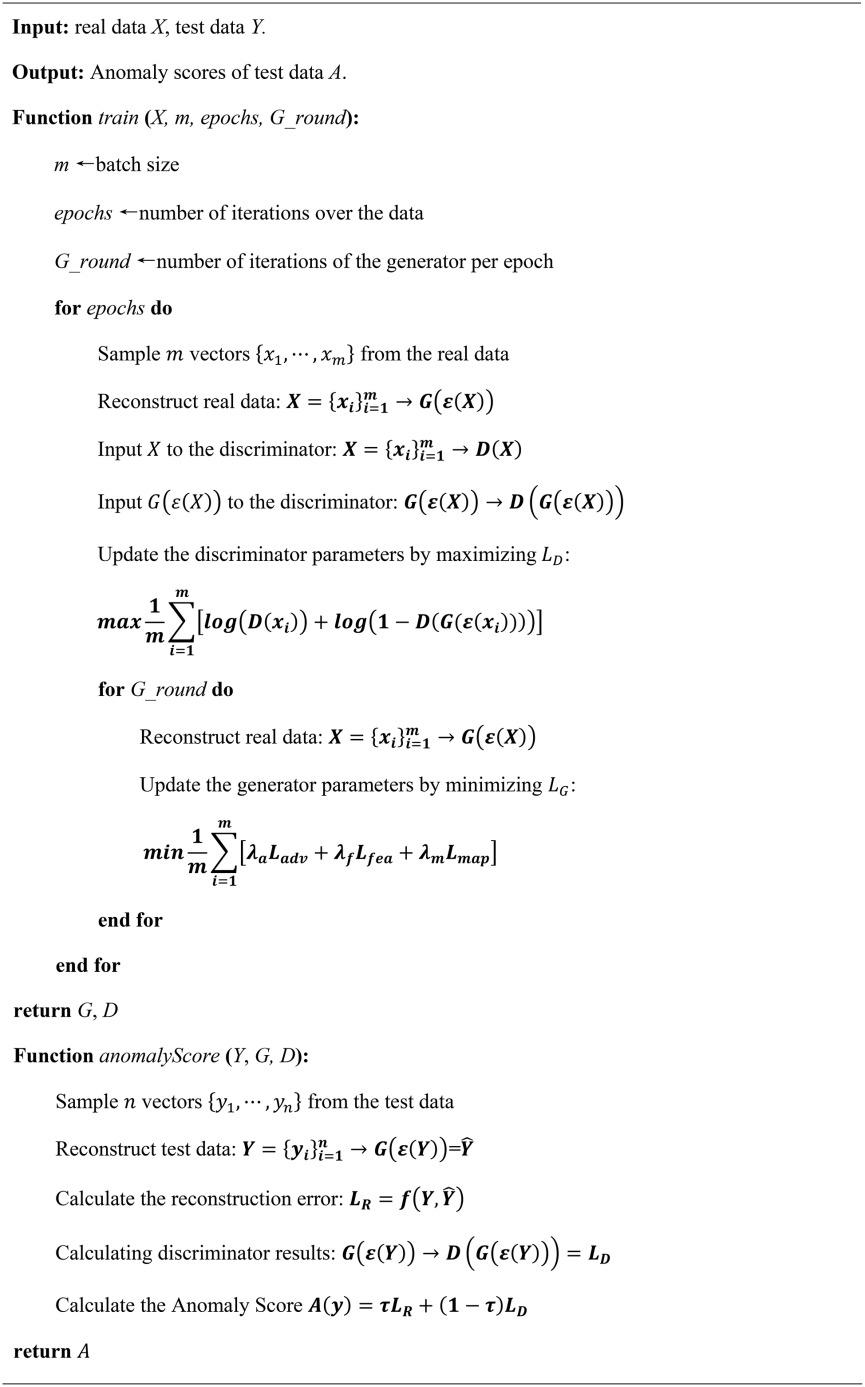
Algorithm for proposed method.

## Experiments

In this section, we present the experimental design and the experimental results. The experimental design contains data set processing and parameter settings. The evaluation metrics for anomaly detection are briefly described. Experimental results include comparison experiments and model performance. Finally, the results of each experiment are discussed.

### Datasets and Experimental Settings

To evaluate the performance of the proposed model, we test it on an agriculture-related time series data set. For future anomalies that may be encountered in smart agriculture, we mainly use three data sets that can represent relevant anomalous behaviors: SWMRU (USDA-ARS, 2016), KDDCUP99 (Blake and Merz, 1999), and HomeC (Taranveer, 2019).

The SWMRU data set contains 15-min mean weather data from the United States Department of Agriculture-Agricultural Research Service (USDA-ARS) Conservation and Production Laboratory (CPRL), Soil and Water Management Research Unit (SWMRU) research weather station, Bushland, Texas (Lat. 35.186714°, Long. -102.094189°, elevation 1,170 m above MSL) for all days in 2016. The data set has 18 variables and 35,139 time durations and it collects the values of sensors deployed at different heights on the grass during the irrigation season.

KDDCUP99 data set is the data set used for The Third International Knowledge Discovery and Data Mining Tools Competition. The data set is a network traffic data set that has 42 variables with 56,235 data points each. This data set is used to train a network intrusion detection model, which is adopted in our experiments to simulate possible anomalies in smart agricultural IoT due to intrusions. Most anomaly detection data sets have far fewer anomalous data points than normal data points, which leads to an imbalance in anomaly detection. This data set is a relatively balanced data set and its introduction allows for a more objective assessment of model performance.

The existing public data set for IoT power monitoring in agriculture has small dimensions, so the smart home IoT power usage data set HomeC can be used to simulate the power monitoring of IoT devices in future smart agriculture. It is collected in a smart home application scenario, and the data structure is similar to that of the agricultural IoT data set but with higher dimensionality. This data set contains 32 variables with 503,900 data points each. The information about the data set is presented in Table 1. The normal data points in the data set are marked as 1 and the abnormal data are marked as 0. The original data set is divided into the training set, the validation set, and the test set, and the training set contains only normal data. We use an unsupervised training approach, where the labeled validation set is used to find the optimal parameters of the model, and the labeled test set is used to compute the results of anomaly detection to evaluate the model performance.

**Table 1:** Details of datasets.

Dataset	Number of variables	The total length of time series	Proportion of anomaly
SWMRU	18	35,139	5%
KDDCUP99	42	56,235	19.5%
HomeC	32	503,900	8%

For data preprocessing, we use a sliding window mechanism to partition the data as described in section 3.1. The optimal window size is an important element in the study of time series data. For this case, we use different window sizes, namely, $s_{w}=30\times i,i=1,2,\cdots 10$ , to capture the state of the data at different accuracies. The results of this experiment are useful for exploring the effect of window size on detection performance. To better capture the normal data distribution, the training phase time step $s_{t}$ is set to 10. During the testing phase, the time step is set to a time window size to ensure that anomalies are not repeatedly detected. The generator uses an Enhanced LSTM as the encoder and decoder, where the Enhanced LSTM depth is set to 3 and the hidden unit is set to 100. Generally, the discriminator follows the same parameter settings. However, unlike the generator, the final output dimension is 1, because the value of the discriminator indicates the degree of abnormality of the input sample. Li et al. (2019) evaluated the effect of latent vector dimensionality on the results in their experiments and verified that a dimension of 15 produced better data reconstruction, so we consider setting the dimension to 15 in our experiments. Since the discriminator converges faster, we set to train the discriminator once in one epoch but train the generator three times, with the epoch set to 100. The main parameters of the model are shown in Table 2.

**Table 2:** Model parameter settings.

Window size	Training window step size	Test window step size	Input dimension	Number of LSTM hidden units	Number of LSTM layers	Latent space dimension
30×i,i=1,2,⋯10	10	Window size	Data set dimension	100	3	15

### Evaluation Measures

We use three standard evaluation measures, namely, Precision (Pre), Recall (Rec), and F1 score, to evaluate the anomaly detection performance of the proposed model:


(14) $$Pre=\frac{TP}{TP+FP}$$

(15) $$Rec=\frac{TP}{TP+FN}$$

(16) $$F1=2\times \frac{\mathrm{Pr}e\times Rec}{Pre+Rec}$$

The objective of the model is anomaly detection, so the detected anomalies are positive samples. Therefore, TP is the correctly detected abnormal (True Positives: detected as abnormal while labeled as abnormal), FP denotes the incorrectly detected abnormal (False Positives: detected as abnormal while labeled as normal), TN represents the correctly detected normal (True Negatives: detected as normal while labeled as normal), and FN means the incorrectly detected normal (False Negatives: detected as normal while labeled as abnormal). TP+FP denotes all the anomalies detected by the model, so precision indicates how many of the detected anomalies contain real anomalies, while TP+FN is all the actual anomalies, so recall indicates how many of all the existing anomalies are detected by the model. The F1 score is the equal-weighted harmonic mean of the precision and recall. In the application scenario of anomaly detection anomalies are not common; that is, the distribution of anomalous and normal data is not balanced. Thus, the accuracy metric will not be used to evaluate the performance of the model.

### Results and Discussion

We evaluate the anomaly detection performance of the proposed model on the above three data sets. To compare the performance of the models, MAD-GAN, TadGAN, TAnoGAN, and AutoEncoder (AE) were adopted to perform experiments on the same data set and record the experimental results. The above four counterpart models commonly used the reconstruction-based anomaly detection methods (Malhotra et al., 2016; Li et al., 2019; Bashar and Nayak, 2020; Geiger et al., 2020). In addition, to verify the validity of our proposed model structure, the results of the ablation experiments are shown and discussed.

### Data Reconstruction Performance

**Figure 7:**
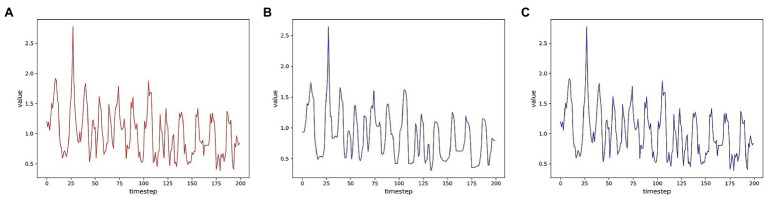
Comparison of reconstruction effects on the SWMRU data set **(A)** Real sample; **(B)** No attention mechanism; **(C)** attention mechanism.

**Figure 8:**
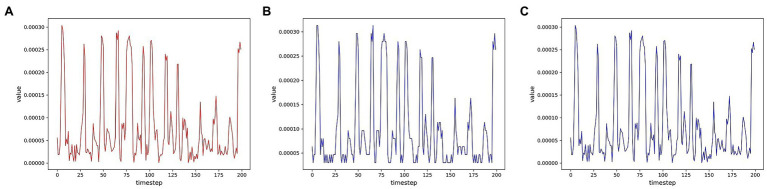
Comparison of reconstruction effects on the HomeC data set **(A)** Real sample; **(B)** No attention mechanism; **(C)** Attention mechanism.

To evaluate the reconstruction ability of the generator for the samples, we first visualize the multidimensional time series samples generated by the model with the original data. For more visualization, only one of these dimensions is shown for two data sets. In order to measure the degree of improvement of the attention mechanism on the reconstruction effect, the samples generated by the models without the attention mechanism are shown together. As shown in Figures 7, 8, the samples without the attention mechanism have largely conformed to the original sample distribution in terms of the overall trend. However, the comparison shows that the attention mechanism still leads to an improvement in the reconstruction effect. When the curve changes more dramatically, the generated samples are closer to the original samples because the attention mechanism allows the model to learn the samples more accurately.

**Figure 9:**
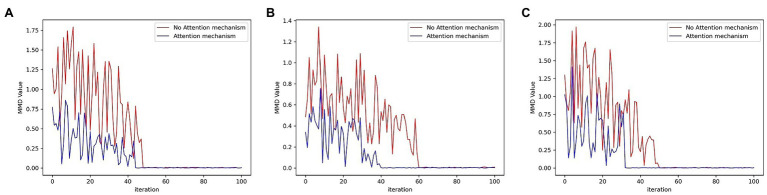
MMD values for each data set **(A)** SWMRU; **(B)** KDDCUP99; **(C)** HomeC.

In addition, Maximum Mean Discrepancy (MMD) was used to evaluate whether the GAN model actually learns the distribution of the training data (Li et al., 2015, 2019). Therefore, MMD was also introduced into the experiment to compare the effect of reconstruction. The decrease in the MMD values indicates that the data generated by the model conform more to the distribution of the original sample. The MMD values generated from the three data sets by iterative training of the GAN are plotted in Figure 9. As shown in these figures, as the number of iterations increases, the model outputs samples that are increasingly closer to the original samples. And the samples generated in the three data sets by the model incorporating the attention mechanism obtained lower MMD values. The MMD value more clearly illustrates that the attention mechanism improves the reconfiguration effect.

### Window Setting and Reconstruction Error Metric

The sliding window size setting is critical to the processing of time series data, so we conduct experiments on the validation data set to determine the appropriate window size. The relationship between the sliding window size setting and the reconstruction error metric will also be discussed in this section. Next, the experiments are described using the HmoeC data set as an example. The sliding window size is still set to $s_{w}=30\times i,i=1,2,\cdots 10$ , but the reconstruction error is calculated in two ways to explore its relationship with the window size. The model proposed in this paper uses the point-wise difference coupled with the DTW algorithm results as the final reconstruction error calculation, where the parameters α and β are derived from multiple experiments on the validation set tuned according to different data sets.

**Figure 10:**
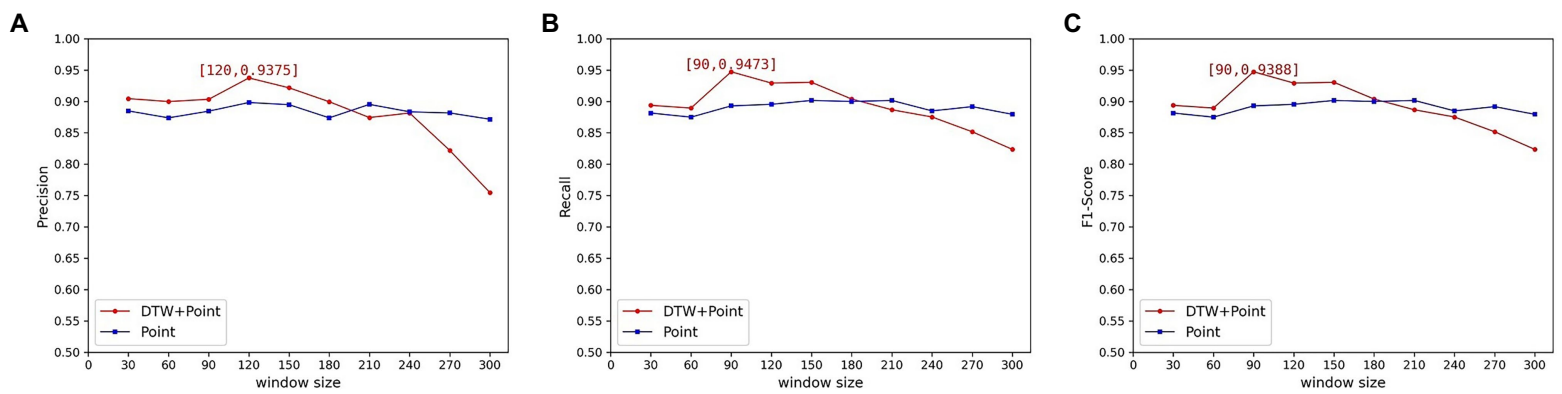
Variation of metric with time window for the HomeC data set**(A)** Precision; **(B)** Recall; **(C)** F1 Score.

In the previous experiments, we used the coupling results as a reconstruction error metric to determine its potential correlation with the window size. It was found experimentally that all three indicators of the experiment showed a decreasing trend as the time window increased. And when the time window increases to a certain extent, these indicators show a large decline. However, the common models that use point-wise error as a reconstruction metric do not show this phenomenon. For comparison, we conducted experiments using the universal point-wise difference calculation ( β = 0). None of the three indicators showed a significant decrease with increasing time windows. The precision, recall, and F1 scores of the data set are shown in Figure 10.

From the above experimental results, it can be seen that the best values of precision, recall, and F1 score can indeed be obtained by using the calculation method proposed in this paper. However, as the time window increased, the experimental results showed a significant downward trend. This is because the DTW algorithm outputs the similarity measure of the two curves, and it is proposed to solve the time shift problem of the curves. But the tolerance of the DTW algorithm to curve similarity increases due to the excessive time window. The DTW algorithm has a small probability of finding the optimal distance between two curves in a small window and outputs a large calculated value. The anomaly will be detected because it improves the reconstruction error value. When the window becomes larger, the DTW algorithm can always find the corresponding minimum distance. Therefore, the output value is reduced, resulting in the abnormal subsequence being incorrectly classified as normal.

The experimental results also clarify that the window size does have a large effect on the results, so the window size needs to be determined reasonably. The window size should be strictly determined when using DTW as a reconstruction error metric. The experimental time window size is finally determined to be 90, at which time the optimal F1 score is 0.9388.

### Comparison Experiments and Discussion

The window size is set to 90 based on the results obtained in section 4.3.2, and the remaining hyperparameters are tuned using the validation set. Anomaly detection is performed on three test data sets using our proposed model with optimal hyperparameters to obtain precision, recall values, and F1 scores. To demonstrate the effectiveness of our proposed model, we conduct experiments using each of the four reconstruction-based anomaly detection models mentioned above, including MAD-GAN, Tad-GAN, TAnoGAN, and AE. The average values of Precision, Recall, and F1 score after ten rounds are calculated on three data sets, and the comparison results of five algorithms are shown in Table 3. As shown in Table 3, the metrics of our proposed model exceed 0.9 on all three data sets and outperform other algorithms in several of the three metrics. The experimental results indicate that the model proposed in this paper has better performance and outperforms other algorithms in the specified data set.

**Table 3:** Experimental results of different methods on three data sets.

Data set	Methods	Precision (%)	Recall (%)	F1 score
SWMRU	Ours	**92.37**	**95.55**	**0.9482**
	Ours-Dis	92.05	94.50	0.9403
	Tad-GAN	91.08	94.13	0.9348
	Ours-Gen	87.95	90.31	0.8891
	Ours-Gen-Dis	87.16	89.94	0.8835
	MAD-GAN	85.41	89.23	0.8754
	TAnoGAN	86.43	89.35	0.8876
	AE	69.48	75.26	0.7238
KDDCUP99	Ours	**93.51**	**96.25**	0.9385
	Ours-Dis	93.38	96.05	0.9365
	Tad-GAN	93.17	94.83	**0.9405**
	Ours-Gen	87.19	92.35	0.8847
	Ours-Gen-Dis	86.49	91.42	0.8794
	MAD-GAN	83.65	89.30	0.8689
	TAnoGAN	85.58	88.23	0.8736
	AE	75.43	81.41	0.7749
HomeC	Ours	**91.12**	**92.79**	**0.9235**
	Ours-Dis	90.73	92.48	0.9205
	Tad-GAN	88.49	92.53	0.9199
	Ours-Gen	85.74	88.63	0.8749
	Ours-Gen-Dis	84.16	88.11	0.8636
	MAD-GAN	83.39	87.09	0.8419
	TAnoGAN	82.24	88.67	0.8676
	AE	67.26	72.84	0.6929

The bold values in Table 3 are the highest values of each experimental metric for each data set.

In addition, to demonstrate the validity of our proposed model improvement, the results of the ablation experiments are also presented in Table 3. To prove the effectiveness of the encoder–decoder architecture containing the attention mechanism, we modify the generator to the same LSTM architecture as MAD-GAN. At the same time, the discriminator and the error calculation method are kept constant. The reconstruction error is obtained after generator inversion. The experimental results are noted as “Ours-Gen,” that is, the experimental results obtained by removing the improvements of the generator. To demonstrate the boosting effect of Enhanced LSTM on the discriminator, we also keep the remaining architecture constant and only change the generator to the general LSTM architecture. Since there is no change in the generator, the experimental results can be obtained directly without generator inversion. The experimental results are noted as “Ours-Dis,” which is the experimental result obtained after removing the discriminator improvement. For the error calculation method, the experimental results are noted as “Ours-Gen-Dis.” After the generator and discriminator improvements are all removed, the remaining architecture of our proposed model is equivalent to MAD-GAN except for the error calculation method. As shown in Table 3, after removing the generator improvements, our proposed model shows a substantial decrease in the experimental metrics for all three data sets. It is concluded that our proposed encoder–decoder architecture incorporating the attention mechanism does improve the model performance. In addition, the results of the “Ours-Dis” also showed a small decrease. The Enhanced LSTM that was introduced into the discriminator is also relevant for model performance improvement. The experimental metrics of “Ours-Gen-Dis” are higher than MAD-GAN, which can prove that our proposed error calculation method improves the detection effect.

The model proposed in this paper significantly outperforms AE, MAD-GAN, and TAnoGAN in all three metrics. Our proposed model generator is similar to AE, but the final detection performance is better than AE. The autoencoder alone does not detect anomalies very well, because the autoencoder trained with appropriate loss functions in adversarial training is better able to learn the general data distribution. The better the generator learns normal data, the more sensitive it is for the abnormal data in anomaly detection. For MAD-GAN and TAnoGAN, these two models share a similar structure, in which their generators are similar to simple decoders. They both use random normal data directly to generate the reconstruction data, after which the reconstruction error is calculated. In order to obtain the accurate reconstruction error, it is necessary to find its corresponding optimal latent vector for the test sample. In the reconstruction error calculation process of the two models mentioned above, the best latent vector is derived from the inversion of the test sample by the generator. This may allow the model to improve the reconstruction performance on the test sample, thus allowing the reconstruction error values to be reduced to the extent that affects the final test results. Our models are trained based on normal samples, and both the encoder and decoder learn the two mappings based on the distribution pattern of the normal samples. The encoder learns how to map a normal sample to a latent vector to reconstruct the normal sample. And for test samples that may have anomalies, the encoder mapping may lose some information. The same is true for the decoder, which learning goal is to improve the reconstruction ability of the latent vector for normal samples. After two mappings, test samples with distribution patterns that differ significantly from the normal sample may yield greater reconstruction errors. In other words, the encoder–decoder structure can widen the gap between normal and abnormal samples, which helps to improve detection performance.

On the other hand, TadGAN introduced the cycle-consistent loss and trained the encoder together with the generator, which was used to learn the mapping of normal data to latent vectors. Both this model and our model train the encoder and generator together, so they have almost similar experimental performance. The difference is that this model used cycle-consistent loss for training and introduced a new discriminator for the encoder to improve learning, whereas our model improves learning through an attention mechanism. Both training methods prevent the contradiction between the encoder and the generator and find the corresponding optimal latent vector to the test sample using the most direct method. TadGAN explored different ways of coupling different reconfiguration computations with discriminator outputs, and we have conducted experiments using its best structure. The average F1 score of this model is higher than that of our model, but the recall of our model is higher than it. This proves that our model can detect more anomalies that are present actually. Meanwhile, the optimal F1 value of our model outperformed it in ten training rounds.

## Conclusion

In this paper, we proposed a GAN-based anomaly detection model for multidimensional time series data generated in smart agricultural IoT. This model used the GAN architecture to learn the distribution patterns of normal data and applied reconstruction methods for anomaly detection. Considering the time dependence of time series data and the potential correlation between multidimensional variables, an improved Enhanced LSTM network to form the basis of the GAN was considered in this model. For the problem of generator inversion, the encoder–decoder architecture was adopted as the generator structure of GAN. The co-training of the encoder and decoder eliminated the inversion of the generator for test samples. This effectively reduced the computation time and met the demand for real-time anomaly detection. The performance of anomaly detection has been improved by the use of encoder–decoder architecture. To further improve the reconstruction effect, the encoder–decoder architecture incorporates an attention mechanism, which can extract weights in the time and feature dimensions to help the model reconstruct the samples. For anomaly detection, a new anomaly score calculation was proposed, which took the coupled result of the point-wise difference error and the curve similarity metric as the reconstruction error. The point-wise error and curve similarity were considered together to better fit the definition of realistic error.

Experiments were designed on three smart agriculture-related data sets and these results were compared with four previous anomaly detection algorithms to verify the effectiveness and superiority of the algorithm. The results proved that our method outperformed other methods in most of the metrics, and the error calculation method proposed in this paper can better detect the anomaly. Not only that, our proposed model obtained superior experimental metrics on high-dimensional smart agriculture data sets, which also reflects that GAN can better handle high-dimensional time series data. With the continuous development of smart agriculture, the dimensionality and quantity of data will grow. The model proposed in this paper also provides a new and useful insight for the anomaly detection of high-dimensional time series data in smart agriculture. However, the time window size setting needed to be considered primarily, which may be the reason why this model is lower than one of the counterpart models in terms of F1 score. Thus, how to choose time windows in time series is an important research topic, and the calculation method proposed in this paper also has a strong correlation with the size of time windows, we will continue our work on anomaly calculation methods and time windows in the future.

## Data Availability Statement

Publicly available datasets were analyzed in this study. These data can be found at: https://archive.ics.uci.edu/ml/datasets/KDD+Cup+1999+Data, https://www.kaggle.com/taranvee/smart-home-dataset-with-weather-information, and https://catalog.data.gov/dataset/data-from-quality-controlled-research-weather-data-usda-ars-bushland-texas.

## Author Contributions

WC: conceptualization, methodology, and supervision. TM: model and experiment design and analysis and writing—original draft preparation. XW: software and writing—editing. GW: simulation, writing—review, and project administration. All authors have read and agreed to the published version of the manuscript.

## Funding

This research was funded in part by the Open Project Fund of Key Laboratory of Mine Disaster Prevention and Control under grant no. SMDPC202102 and supported in part by the Graduate Research and Practice Projects of Minzu University of China.

## Conflict of Interest

The authors declare that the research was conducted in the absence of any commercial or financial relationships that could be construed as a potential conflict of interest.

## Publisher’s Note

All claims expressed in this article are solely those of the authors and do not necessarily represent those of their affiliated organizations, or those of the publisher, the editors and the reviewers. Any product that may be evaluated in this article, or claim that may be made by its manufacturer, is not guaranteed or endorsed by the publisher.
